# Comparative Insights into the Fundamental Steps Underlying Gelation of Plant and Algal Ionic Polysaccharides: Pectate and Alginate

**DOI:** 10.3390/gels8120784

**Published:** 2022-11-29

**Authors:** Sergio Paoletti, Ivan Donati

**Affiliations:** Department of Life Sciences, University of Trieste, Via L. Giorgieri 4, I-34127 Trieste, Italy

**Keywords:** alginate, pectate, condensed and bound ions, egg-box model

## Abstract

Pectate and alginate are among the most important biopolymers able to give rise to ionotropic gelation upon the addition of di- or multivalent counterions. The two ionic polysaccharides exhibit several common aspects of the gelation mechanism with calcium ions, the physiologically and commercially most relevant counterion type. The first one pertains to the role that specific Ca^2+^/polyion interactions play in the establishment of the ion-mediated chain/chain cross-links. Such interactions include both a specific affinity of the territorially condensed Ca^2+^ counterions for the polyuronate(s) and the formation of long-lasting chemical bonding (inner ion–sphere complex) of specific interchain sites accompanied by high conformational ordering. As to the first mechanism, it is dominated by the strong desolvation of the interacting ionic species, with concomitant positive variations in both enthalpy and entropy, the contribution of the latter prevailing over the former due to the favorable liberation of a very large number of water molecules of hydration. Both dilatometric and microcalorimetric data point to the higher affinity of Ca^2+^ for pectate than for alginate. The selective accumulation of calcium ions close to the polyanion(s) favors the onset of the second—chemical bonding—mode, which is associated with charge neutralization at the bonding site. This mode coincides with the largely accepted “egg-box” model for the calcium-mediated interchain junction of pectate and alginate. A new approach was devised for the calculation of the fraction of chemically bound divalent ions; it was based on the available circular dichroism data (further supported by scattering and viscosity results) and successfully tested by comparison with an independently determined fraction in the case of pectate. In detail, the strong bonding mode manifests in two sequential bonding modes. The first one (at low concentrations of added Ca^2+^ ions) entails a cross-link in which only one calcium ions is bracketed in a “twisted” egg-box between two chains; upon further counterion addition, a series of nearest-neighboring “perfect” egg-box structures develops. Both dilatometric and microcalorimetric changes associated with the latter chemical bonding modes are quantitatively larger for pectate than for alginate; clearly the latter polyuronate suffers from the relevant presence of the weakly calcium-binding mannuronic acid repeating units. Light-scattering experiments provided a clear-cut demonstration of the intermolecular bonding of calcium ions from the very beginning of the linker addition.

## 1. Introduction

Among biopolymers, polysaccharides have certainly turned out to be the most versatile in giving rise to both chemical and physical gels. In the first case, the wealth of functional groups (mostly the hydroxyl group but also the amino group) lent themselves to a range of cross-linking chemical reactions, creating covalent bonds. Chemically cross-linked dextran proved to be a real breakthrough in separation science and technology [1], while—more recently—chemical cross-linking reactions produced cellulose-based gels for application as biomaterials [2,3]. Polysaccharide physical gels, however, have been much more successful, reaching unsurpassed application in the food, pharmaceutical, and biotechnological fields. Both neutral (e.g., amylose, starch, agar) and ionic (e.g., alginates, pectins, carrageenans, xanthan, gellan gum) carbohydrate polymers played a pivotal role in the quoted fields, being worth billions of USD on the global market [4]. Their success is rooted in the molecular mechanism of formation:

“Physical gels are characterized by dynamic cross-links that are constantly created and broken, changing its state between solid and liquid under influence of environmental factors. This restructuring ability of physical gels makes them an important class of materials with many applications, such as in drug delivery”.

[5]

The goal of this paper is to highlight similarities and difference between two commercial sample of polyuronates that find applications in several fields as to their ability to bind calcium ions to eventually give rise to ionotropic gels, namely sodium pectate [6,7] and sodium alginate [8]. The former sample is a highly de-esterified pectin, whereas the latter sample is a typical “gelling” alginate, extracted from the stipes of the brown seaweed *Laminaria hyperborea* (*L. hyp.*), rich in the high calcium-binding component guluronic acid (GulA) (see Section 4). To this end, focus will be given to different physical–chemical aspects, from the thermodynamic ones to those more directly pertaining to their macromolecular behavior, all being rooted in the divalent counterion–carbohydrate polyion interactions.

The assessment of the fundamental role of contributions of different nature (specific ionic affinity, intimate ion pairing with charge annihilation) [9,10] to the overall binding ability will require an estimate of the extent of each ion-binding mode. A thorough analysis of the thermodynamics of calcium binding by pectate was recently published using a combination of chiro-optical and microcalorimetric data [11]. Such combination of experimental approaches, together with a non-trivial minimization process based on previous theoretical studies [9,10,11], proved to be a robust method for the complete description of the thermodynamics of divalent counterion-binding by pectate. In addition to giving quantitative data on binding, it provided values for the (microscopic) changes of Gibbs free-energy, enthalpy, and entropy, for both specific affinity territorial bonding and strong inner-sphere complex formation. However, it was desired to devise a simple, alternative approach based on the faster-to-collect chiro-optical data alone. A new method is herein presented, with the limited goal of providing the values of the fraction of chemically bonded ions upon increasing the concentration of the added divalent ion; it will initially be tested by comparing its predictions with those from the more complete one for the case of pectate [11], and then applying them to alginate. Knowledge of the fractions will turn out to be crucial for the full exploitation of the information deriving from macromolecular techniques, namely viscosity and light scattering. At the end, a significant difference will be revealed in the development of the—basically identical—interchain Ca^2+^ bonding between the gelling polymer of vegetal origin (pectate) and that of algal origin (alginate). The second element of novelty deals with the finding that the thermodynamic parameters of the specific interaction of the divalent ion Mg^2+^ with various polyuronates (namely pectate, algal alginate, the related homopolymer poly(guluronate), polyG, and the regularly alternating copolymer polyMG—M standing for mannuronate) can be successfully accounted for by a linear enthalpy/entropy correlation, thus pointing to a common underlying mechanism governing the interaction with different polyuronates.

## 2. Results and Discussion

The comparison of the calcium-binding behavior of pectate with that of alginate will start by briefly reviewing the data of the former polyuronate already published [11,12].

### 2.1. Pectate

**Scheme 1:**
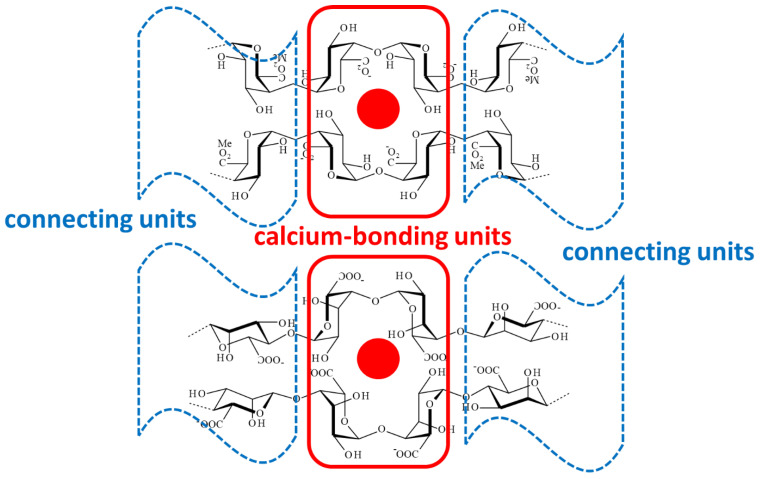
Idealized representation of the calcium-binding site of pectate (**upper** part) and alginate (**lower** part) in the so-called “egg-box” configuration. The anionic repeating units involved are α-D-galacturonate and α-L-guluronate, respectively (red rectangle). The repeating units connecting the binding sites are the methyl-ester forms of α-D-galacturonate and β-D-galacturonate units, respectively (dotted blue waves). Ca^2+^ ions are represented as red full circles.

The interaction of calcium ions with pectate (or poly(galacturonate)) was shown to be consistently traced back to two major types, namely the affinity one and the chemical bonding one. Within the framework of the counterion condensation (CC) theory of linear polyelectrolytes [13,14], extended to encompass the case of systems containing counterions of mixed valency [15], further theoretical developments included the specific affinity that condensed divalent counterions may exhibit toward the polyanion [10]. Finally, the strong chemical bonding—albeit not a covalent one—was also successfully included in the theoretical extension [9]. It may arise from an intimate interaction of the ionic species with the opposite sign, up to the level of charge annihilation at least as perceived on the scale of polyelectrolyte interactions (Debye length). Inner-sphere (or “contact”) ion pairs, in which there is no water molecule to separate the ions of the pair, are a suitable example (e.g., see references [16,17]). The application of the above theoretical treatments lead to a successful description of the interaction of Ca^2+^ ions with pectate [12]. The analysis was lately extended to obtain a full description of the thermodynamic parameters of that interaction, based on chiro-optical, microcalorimetric, dilatometric, viscometric, and molecular-weight-determining methods [11]. That paper convincingly demonstrated that the strong chemical bonding of calcium by the coordinating groups of poly(galacturonate) (namely the carboxylate group and the ring hydroxyl ones) perfectly matched the two-type model of bonding that had been previously set forth to explain the behavior of Ca^2+^/alginate system [18]. That model envisages an intermolecular chelation of the alkaline-earth ion by coordinating groups on two facing polyanion chains. Ions are initially bonded in a “twisted” egg-box mode (bonding type-1), allowing the four polyanionic stretches departing from the cross-link of the two alginate chains to assume the farthest relative distance because of the strong electrostatic repulsion. Further bonding produced by an increase of added calcium ions then brings about the formation of sequences of neighboring calcium ions in an arrangement that can be more or less strictly resemble that of the perfect “egg-box” model set forth as long as almost fifty years ago by the group of D.A Rees [19] (bonding type-2)—see Scheme 1. (Actually, the thorough modeling work by Braccini and Pérez indicated that, in the case of pectate, a relative shift of the two—antiparallel—facing chains better conforms to energy calculations, but not in poly(guluronate)) [20]. More recently, a convincing fiber X-ray diffraction study on calcium–alginate gels provided direct structural evidence of the “egg-box” model [21]. Interestingly, the fundamental steps of calcium bonding by pectate and alginate (that are at the root of the eventual formation of calcium–polyuronate gels) perfectly conform to the predictions of the theory developed by Borukhov et al. to describe the association of two semiflexible polyelectrolytes by interchain linkers [22].

Reference [11] allowed for providing values for the fundamental thermodynamic properties of both affinity and the two modes of strong chemical bonding, which have been herein reported in Table 1.

**Figure 1:**
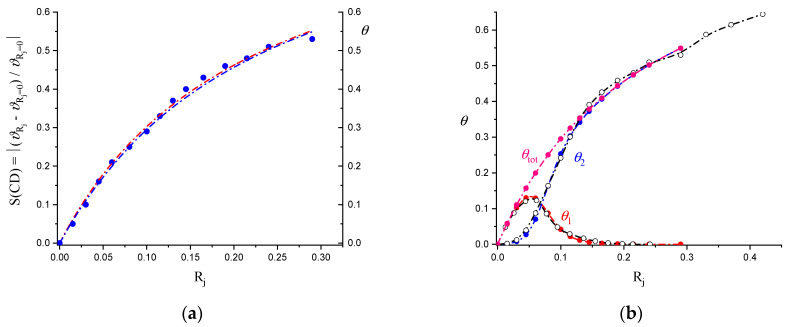
(**a**) Dependence of the absolute value of the “specific” change of the molar ellipticity of sodium pectate as a function of the calcium-to-polymer molar ratio R_j_. Blue full circles, experimental data (monomodal); blue dash-dotted curve, fitting curve with the present method; red dash-dotted curve, fitting with the Langmuir model. (**b**) Fractions of chemically bound calcium ions by sodium pectate as a function of the molar ratio R_j_ calculated from the fitting of the CD data of panel (**a**). Red symbols, fractions of bonding mode 1, $\theta_{1}$, blue symbols, fractions of bonding mode 2, $\theta_{2}$. The open black circles and the dash-dotted black curves correspond to the values of the fractions for each type of bonding as calculated and reported in reference [11].

Moreover, that treatment was able to produce the values of the amount (as fractions: $\theta_{1}\left(R_{j}\right)$, $\theta_{2}\left(R_{j}\right)$) of bound calcium in the different modes, as a function of the increasing amount of added divalent counterions. The latter results have been replotted in panel (b) of Figure 1 (black symbols) as a function of added Ca^2+^ ions as the independent variable, conveniently expressed as the molar ratio of calcium to polymer (repeating units), R_j_.

### 2.2. Alginate

In the case of alginate, the knowledge of the R_j_—dependence of the fractions of chemically bonded calcium ions from the thorough analysis of chiro-optical and microcalorimetric data as that performed on pectate is lacking. We then resorted to constructing a new model to describe bonding, which will be initially tested using the above $\theta_{1}\left(R_{j}\right)$, $\theta_{2}\left(R_{j}\right)$ data of pectate.

#### 2.2.1. Description of the Model

**Scheme 2:**
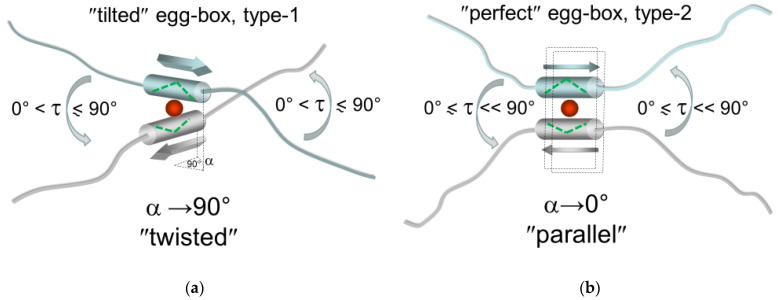
The two types of chemical bonding of Ca^2+^ by polyuronates: (**a**) the “tilted” egg-box bonding mode, (**b**) the “perfect” egg-box bonding mode.

The derivation of the model equations started assuming that the $\theta_{i}$ (i = 1,2) vs. $R_{j}$ dependence can be treated with a model of the adsorption of calcium by the polyuronate “surface” onto a given total number of sites; however, these sites can bind calcium ions according to two modes, as indicated for pectate by the previous results [11], and for alginate by the bimodal behavior of the chiro-optical data (see Figure 1b): a first adsorption type (type-1) corresponding to the “initial” binding (“tilted” egg-box, Scheme 2a and a second adsorption type (type-2) corresponding to the formation of geometrically (or conformationally) ordered egg-box structures (“perfect” egg-box, Scheme 2b). In practice, the two modes of binding correspond to two types of sites that are interconvertible (following a cooperative process) [23], in the sense that type-1 switches to type-2 upon reaching a specific $R_{j}$ value that mainly depends on the molecular features of the polyuronates considered (and on all other relevant physical chemical parameters like polymer and counterion concentration, ionic strength, temperature). In a purely phenomenological approach, our goal is to derive modified Langmuir equations for the two types of competitive adsorption to obtain equations for the two sets of fractions of occupancy.

For the case of more than one type of competitive sites, the general Langmuir equation reads:

(1) $$\theta =\underset{i}{\sum }\theta_{i}=\underset{i}{\sum }\frac{K_{a,i\;}C_{j}}{1+\sum_{i}K_{a,i}{}_{\;}C_{j}}$$

where *θ* is the (total) fractional site occupation (*fso*) defined as the ratio of the number of bound sites, *N*, over the total number of sites, *N*_0_, and $\theta_{i}$ is the *fso* for the site of type *i*. Moreover, $\theta_{tot}=\underset{i}{\sum }\theta_{i}$. (It should be recalled that the total number of bonding sites, in general, differs from the total number of polymer repeating units, i.e., the stoichiometric ratio of calcium-per-bonding site, $\sigma^{0}$, is different from 1. In the case of the egg-box model, $\sigma^{0}$ = 0.25, whereas 0 ≤ *θ* ≤ 1. Therefore, the ratio of bound sites over the total number of polymer repeating units, $\sigma_{tot}$, is $\sigma_{tot}=\sigma^{0}\;\cdot \;\theta_{tot}$). $K_{a,i}$ is the adsorption constant of the ligand to the site of type *i,* and $C_{j}$ is the equilibrium (total) concentration of adsorbate (in the present case, the calcium counterion). $K_{a,i}$ is defined as the ratio (quotient) of the adsorption rate for the site of type *i* to the corresponding rate of desorption. To stress that $K_{a,i}$ is just a rate ratio (quotient) and not an equilibrium constant, in the following, it will be indicated as $Q_{i}$. For the present case of two sites, Equation (1) reads:

(2) $$\theta \left(Ca^{2+}\right)=\theta_{1}\left(Ca^{2+}\right)+\theta_{2}\left(Ca^{2+}\right)=\frac{Q_{1\;}\cdot \;\left[Ca^{2+}\right]}{1+Q_{1\;}\cdot \left[Ca^{2+}\right]+Q_{2\;}\cdot \;\left[Ca^{2+}\right]}+\frac{Q_{2\;}\cdot \left[Ca^{2+}\right]}{1+Q_{1\;}\cdot \left[Ca^{2+}\right]+Q_{2\;}\cdot \;\left[Ca^{2+}\right]}=\;=\frac{Q_{1\;}\cdot R_{Ca^{2+}}}{C_{p}{}^{-1}+Q_{1\;}\cdot R_{Ca^{2+}}+Q_{2\;}\cdot \;R_{j}}+\frac{Q_{2\;}\cdot R_{Ca^{2+}}}{C_{p}{}^{-1}+Q_{1\;}\cdot R_{Ca^{2+}}+Q_{2\;}\cdot \;R_{j}}$$

with:

(3) $$\left[{\mathrm{Ca}}^{2+}\right]=R_{j}\;\cdot C_{p}$$

$C_{p}$ being the polyuronate concentration in mol ·L^−1^ (uronate repeating units), and the numerical indexes *i* referring to the “egg-box” type of binding sites, either “tilted” (type-1, “1”) or “perfect” (type-2, “2”).

The following assumptions hold in the derivation of Equation (2):

- there is only one type of adsorbate (i.e., Ca^2+^ ions) and two type of sites with a different affinity for the adsorbate;
- all sites of type *i* show the same value of the quotient $Q_{i}$;
- the bound species attach to definite association sites;
- each site can accommodate only one bound ion;
- there are no interactions between adsorbate molecules on adjacent sites.

The above point *e*. obviously depicts an ideal (but unrealistic) situation; on the contrary, one should take into account not only the strong electrostatic counterion/polyion interactions (Ca^2+^/polyuronate) but also the polyion/polyion ones, assumed to be at the root of the Borukhov approach [22]. Those interactions clearly depend on the linear charge density of the polyuronates, on the dielectric constant ϵ of the solvent, on temperature, on the ionic strength of the solution, on the polymer concentration, and, most importantly, on the calcium-to-polymer molar ratio, $R_{j}$. It then becomes necessary to modify Equation (2) to account for the experimentally observed dependence of (intrinsic) binding parameters on $R_{j}$, which amounts substituting $Q_{1}$ with a calcium-dependent value $Q_{1}\left(R_{j}\right)$. To this end, we resorted to introducing into the above formalism the empirical approach:

(4) $$Q_{1}\left(R_{j}\right)=Q_{1}\;\cdot \;\varphi_{1\;}\left(R_{j}\right)$$

and

(5) $$Q_{2}\left(R_{j}\right)=Q_{2}\;\cdot \;\varphi_{2\;}\left(R_{j}\right)$$

i.e., assuming that both $Q_{1}$ and $Q_{2}$ are constant and independent of $R_{j}$ and that all the corresponding calcium-concentration dependent (non-ideal) terms are collectively expressed by $\varphi_{1\;}\left(R_{j}\right)$ and $\varphi_{2\;}\left(R_{j}\right)$, respectively. (It is much like the electrostatic term $e^{\frac{\Delta G^{el}\left(\alpha \right)}{RT}}$ in the expression of the apparent dissociation constant of a weak polyacid: $K_{diss}^{app}\left(\alpha \right)=K_{diss}^{0}\;\cdot \;e^{\frac{-\Delta G^{el}\left(\alpha \right)}{RT}}$, with α the degree of dissociation). For simplicity and with no loss of clarity, in the following $\varphi_{1\;}\left(R_{j}\right)$ and $\varphi_{2\;}\left(R_{j}\right)$ will be operationally short-named only as $\varphi_{1\;}$ and $\varphi_{2}$, respectively, obviously keeping in mind that they both depend on $R_{j}$.

Equation (2), for the two types of sites, would then be expressed as:

(6) $$\theta_{1}\left(R_{j}\right)=\frac{Q_{1}\;\;\cdot \;\varphi_{1}\;\cdot \;R_{j}}{C_{p}{}^{-1}+Q_{1}\;\cdot \;\varphi_{1}\;\cdot \;R_{j}+Q_{2}\;\cdot \;\varphi_{2\;}\;\cdot \;R_{j}}$$

(7) $$\theta_{2}\left(R_{j}\right)=\frac{Q_{2}\;\cdot \;\varphi_{2}\;\cdot \;R_{j}}{C_{p}{}^{-1}+Q_{1}\;\cdot \;\varphi_{1\;}\;\cdot \;R_{j}+Q_{2}\;\cdot \;\varphi_{2}\;\cdot \;R_{j}}$$

The task at hand is now to obtain expressions for $\varphi_{1}$ and $\varphi_{2}$ that satisfy some clearly identified conditions, albeit arbitrary, herewith listed:

1. *φ*_1_ and *φ*_2_ are mutually interdependent;
2. *φ*_1_ and *φ*_2_ will both vary between 0 and 1, thereby being assumed as purely modulating the “intrinsic” binding affinity of type-1 and type-2 sites through *Q*_1_ and *Q*_2_, respectively;
3. sequentially, the type-1 mode of binding is the first to take place, followed by an interconversion to type-2 mode;
4. the lowest number of parameters has to be used.

In practice, it was decided to write interdependent expressions for $\varphi_{1}$ and $\varphi_{2}$, both being a function of the independent variable $R_{j}\;$($R_{j}=\left[{\mathrm{Ca}}^{2+}\right]/{\;C}_{p}$) with two arbitrary parameters, namely $R_{j}^{Crit}$, the critical value of $R_{j}$ at which $\varphi_{1}=\varphi_{2}$, and τ, an adjustable parameter (front factor) that numerically modulates the transition between the type-1 and type-2 binding modes, arbitrarily choosing the following expressions:

(8) $$\varphi_{1}\left(R_{j}\right)=\frac{e^{\tau \;\cdot \;\left(1-\frac{R_{j}}{R_{j}^{Crit}}\right)}}{1+e^{\tau \;\cdot \;\left(1-\frac{R_{j}}{R_{j}^{Crit}}\right)}}$$

(9) $$\varphi_{2}\left(R_{j}\right)=\frac{e^{-\tau \;\cdot \;\left(1-\frac{R_{j}}{R_{j}^{Crit}}\right)}}{1+e^{-\tau \;\cdot \;\left(1-\frac{R_{j}}{R_{j}^{Crit}}\right)}}$$

$R_{j}^{Crit}$ is not a fitting parameter. In fact, by setting $R_{j}\;\equiv \;R_{j}^{Crit}$ one easily finds that:

(10) (θ1θ2)RjCrit=Q1Q2

which will be used in the minimization cycles. It can be convenient to analyze the limiting behavior of $\varphi_{1}$ and $\varphi_{2}$ as a function of the independent variable $R_{j}\;$and of the parameters $R_{j}^{Crit}$ and τ. The results are reported in Table 2. 

The effect of changing some key parameters on $\varphi_{1}$ and $\varphi_{2}$ is reported in Figure 1.

Figure 1a shows that, upon increasing τ at equal $R_{j}^{Crit}$, the transition between $\varphi_{1}$ and $\varphi_{2}$ is more marked in relative terms. The case reported in Figure 1b is of particular interest. Non-constant values of τ, i.e., τ = τ ($R_{j}$), were used in Equations (8) and (9), corresponding to the full squares in Figure 1b, starting from τ = 9 and progressively decreasing to τ = 0.2. The ensuing initial parts of both $\varphi_{1}$ and $\varphi_{2}$ curves are completely superimposable with the case of constant τ (τ = 9) until about $R_{j}^{Crit}$; after that point, a convergence of the two binding modes takes place. In this sense, the parameter τ can be interpreted as a parameter modulating the observed cooperativity. For the first additions of Ca^2+^, the value of $\varphi_{1}$ is close to 1, and, correspondingly, $\varphi_{2}$ is close to 0; thus, for the very first additions of the cross-linking ion, in practice only the type-1 binding takes place. The sigmoid decrease of $\varphi_{1}$ is very rapid for $R_{j}$ close to $R_{j}^{Crit}$ and progressively smoother thereafter; $\varphi_{2}$ shows symmetrical and opposite behavior.

Further details of the model and of the effect of various parameters on the fitting are given in the Appendix A.

#### 2.2.2. Test of the Model

For each polyuronate, the chemical parameters of interest are the fractions of two bonding types, namely $\;\theta_{1}\left(R_{j}\right)$ and $\theta_{2}\left(R_{j}\right)$ (see Equations (6) and (7)). Their values depend on four “bonding” parameters: $Q_{1}$, $Q_{2}$, τ, and $R_{j}^{Crit}$, whose value can be determined as described. Even if no physical meaning is attributed to each of them, the set of the parameters is obviously expected to change by changing polyuronate but, on the contrary, to be constant for a given polymer when applied to the experimental data from different techniques. For each polymer and each type of experimental technique, the simplest hypothesis is that the experimental data can be fitted with a set of two scaling parameters, one for each type of bonding, assuming that the “intrinsic” relative change of the given property of each of the two modes of bonding is independent of $R_{j}$. Each parameter is the intensive property of the given technique pertaining to the given type of bonding. 

Already in previous work it was found convenient to define the relative change (“specific”, S) of a given physical property Y at a given value of $R_{j}$ with respect to that property at $R_{j}$ = 0, i.e., in the absence of calcium:


(11) $$S\left(Y\right)=\frac{Y_{R_{j}}-Y_{R_{j}=0}}{Y_{R_{j}=0}}$$

The general form of the fitting equation will then read:

(12) $$S\left(Y\right)\left(R_{j}\right)=\Delta A_{Y}\;\cdot \theta_{1}\left(R_{j}\right)+\Delta B_{Y}\;\cdot \theta_{2}\left(R_{j}\right)$$

where $\Delta A_{Y}$ and $\Delta B_{Y}$ are the intensive property values (as fractional changes with respect to the calcium-free case) for type-1 and type-2 bonding modes, respectively. For a single set of Y data for a given polymer, the number of parameters will be five, which is certainly not a small number. Nevertheless, it will be shown that a reasonable test of the model can still be carried out using both the data of pectate and those of alginate.

(a) Pectate

The chiro-optical data of pectate [12] have been reported in Figure 1a. The peculiar feature of the data is that they show a clear monomodal dependence on R_j_ that could be easily described by the Langmuir equation according to a single bonding mode of calcium to pectate. The best-fit parameters of the Langmuir curve are: (V_max_ =) ΔB_[ϑϑ]_ = 0.98 ± 0.05 and *Q*_*ass*_ = 1286 ± 1. Those values compare very well with those reported in the Supplementary Information part of reference [11]: ΔB_[ϑ]_ = 0.98 and *Q*_*ass*_ = 1232 ± 1; it should be recalled that, in the latter case, four more points (from calorimetry) were included.

**Figure 2:**
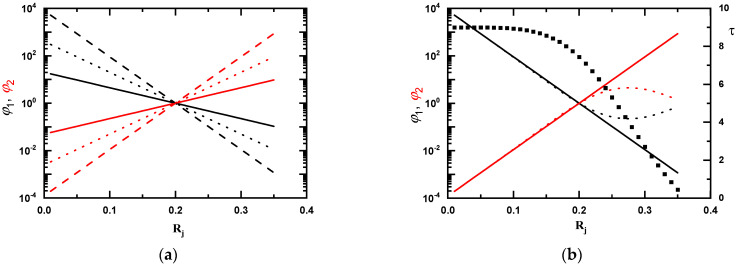
Dependence of $\varphi_{1}$ (Equation (8), black) and $\varphi_{2}$ (Equation (9), red) on $R_{j}$. (**a**) Effect of τ equal to 3 (continuous line), 6 (dotted line), and 9 (dashed line), respectively. ($R_{j}^{crit}$ = 0.2). (**b**) Effect of constant τ (τ = 9, continuous straight lines) or of varying τ(dotted curves) dependence of τ on $R_{j}$ reported on the right y-scale: (**-—-**) constant τ, (■) variable τ.

However, the microcalorimetric data reported in the latter quoted work demonstrated that two different bonding modes (type-1 and type-2) were actually present modulating the calcium–pectate interaction as a function of R_j_. Therefore, two curves, describing the $\theta_{1}\left(R_{j}\right)$ and the $\theta_{2}\left(R_{j}\right)$ dependence on R_j_, were available: they have been reported as dash-dotted black curves in (panel b) of Figure 2. The apparent inconsistency between the CD and the microcalorimetric results as to the mono- or bimodality of curve development as a function of R_j_ was already discussed in reference [11]. It suffices here to recall that, in a two-step process, in which there is an initial conformational ordering that is followed—in the second step—by any rearrangement (like bare lateral association) of ordered chains, one must expect a change of CD in the first step only, whereas changes in the (internal) energetics are likely to take place, with different intensive values, in both steps. We then resorted to testing the model calculating the parameters of best fit—Equations (6)–(9)—using the experimental CD data-points of pectate—namely the ${\left(S\left(\mathrm{CD}\right)\left(R_{j}\right)\right)}^{exp}$ data from panel (a) of Figure 2—and then comparing the values of the fractions $\theta_{1}\left(R_{j}\right)$ and $\theta_{2}\left(R_{j}\right)$ from the new method with those from the previous work [11] (panel b) of Figure 2. (For CD measurements, the absolute value of r.h.s of Equation (11) is used to get rid of the complex interplay of the direction of change (sign) of the molar ellipticity [ϑ] and the sign of [ϑ] at $R_{j}$ = 0 [11]) The calculated fractional change of molar ellipticity ${\left(S\left(\mathrm{CD}\right)\left(R_{j}\right)\right)}^{calc}$ values were computed using the equation:

(13) $${\left(S\left(\mathrm{CD}\right)\left(R_{j}\right)\right)}^{calc}=\Delta A_{\left[\vartheta \right]}\cdot \;\theta_{1}{\left(R_{j}\right)}^{calc}+\Delta B_{\left[\vartheta \right]}\cdot \;\theta_{2}{\left(R_{j}\right)}^{calc}$$

where $\theta_{1}{\left(R_{j}\right)}^{calc}$ and $\theta_{2}{\left(R_{j}\right)}^{calc}$ were obtained by fitting Equations (6)–(9), with the three parameters as to bonding: $Q_{1}$, $Q_{2}$, and τ, further weighted by two scaling parameters $\Delta A_{\left[\vartheta \right]}$ and $\Delta B_{\left[\vartheta \right]}$, recalling Equation (10) for the recursive calculation of $R_{j}^{Crit}$. The best fit was determined by minimizing the error between the experimental ${\left(S\left(\mathrm{CD}\right)\left(R_{j}\right)\right)}^{exp}$ values and the calculated fractional change of molar ellipticity ${\left(S\left(\mathrm{CD}\right)\left(R_{j}\right)\right)}^{calc}$ values:

(14) $$MIN_{CD}=\frac{1}{n}\;\cdot \;\Sigma_{i=1}^{n}{\left({\left(S\left(\mathrm{CD}\right)\left(R_{j}\right)\right)}^{exp}-{\left(S\left(\mathrm{CD}\right)\left(R_{j}\right)\right)}^{calc}\;\right)}^{2}$$

Considering the methodology followed, i.e., a purely phenomenological model rather than a detailed quantitative physical–chemical modeling of counterion–polyion interaction, it is to be recalled that the parameters $Q_{1}$, $Q_{2}$, and τ are devoid of a clear physical meaning; the only goal is to obtain a satisfactory fitting of the experimental CD curves in terms of the two fractions of bound ions to site 1 and 2, respectively. At this stage, only the scaling parameters $\Delta A_{\left[\vartheta \right]}$ and $\Delta B_{\left[\vartheta \right]}$ can bring about physical meaning, providing an indication about the fractional conformational change of the different polyuronates upon calcium binding in the two binding modes, in a comparative approach.

The calculated curves after minimization have been reported in Figure 2, panel a, for ${\left(S\left(\mathrm{CD}\right)\left(R_{j}\right)\right)}^{calc}$, and in panel (b) for ${\left(\theta_{1}\left(R_{j}\right)\right)}^{calc}$ and the ${\left(\theta_{2}\left(R_{j}\right)\right)}^{calc}$, respectively. Panel (b) also reports the experimental $\theta_{1}\left(R_{j}\right)$ and $\theta_{2}\left(R_{j}\right)$ data points from reference [11] as black dash-dotted curves. The parameters of interest are: $Q_{1}$ = 1180, $Q_{2}$ = 1200 (i.e., $Q_{1}$ ≈ $Q_{2}$); ΔA_[ϑ]_ = 1.01_5_, ΔB_[ϑ]_ = 1.01_7_ (i.e., ΔA_[ϑ]_ ≈ ΔB_[ϑ]_), respectively (τ is attributed no physical meaning). The mean squared error (MSE) is: 1.84E-04, with MSE = RSS/(n − p), n = data points, p = number of parameters (5: Q1, Q2, ΔA_[ϑ]_, ΔB_[ϑ]_, τ), and RSS = residual sum of squares. r.m.s. error = 0.01_4_E-02. The estimated experimental error on ${\left(S\left(\mathrm{CD}\right)\left(R_{j}\right)\right)}^{exp}$ is 0.02.

The results are quite encouraging: the fact that $Q_{1}$ ≈ $Q_{2}$ to within 1.6% is reflected in the value of their ratio: Q1Q2 = 0.98_3_ (i.e., −1.7% from 1). According to Equation (10), (θ1θ2)RjCrit should be equal to ≈ 1: interestingly, the value of $R_{j}^{Crit}$ at which $\theta_{1}$ ≈ $\theta_{2}$ is 0.068_5_ from the $\theta_{1}$, $\theta_{2}$ plot with the new method, whereas it is 0.066_9_ from the plot with the data of reference [11]. The present result is only 2.4% lower than the reference one. 

**Figure 3:**
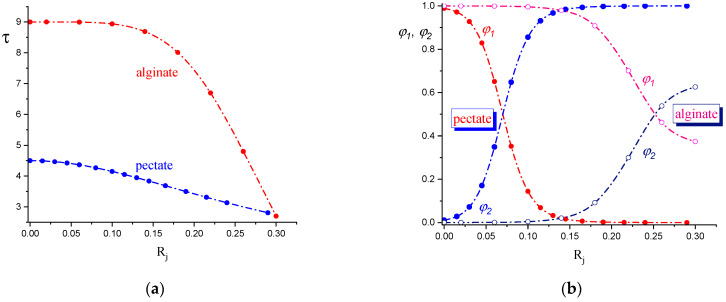
Dependence on the calcium-to-polymer molar ratio Rj of the parameter τ (**a**), and of the parameters $\varphi_{1}$ and $\varphi_{2}$ (**b**) for alginate (upper, red curve in (**a**)) and pectate (lower, blue curve in (**a**)). The colors of the polymer labels (text and frame) in panel (**b**) correspond the relative curves, respectively. The values have been determined according to the procedure (and using the numerical values of the parameters) reported in the Appendix A.

Additionally, the average of $Q_{1}$ and $Q_{2}$ falls within about 6.5% of the two values from the single Langmuir fits. ΔA_[ϑ]_ ≈ ΔB_[ϑ]_ to within 0.1%, and their average falls within about 3% of the values from the two values from the single Langmuir fits (i.e., 0.98). Altogether, those findings should be reasonably taken as a safe indication that $Q_{1}$ = $Q_{2}$ = *Q*_*ass*_ = 1225 ± 46 and ΔA_[ϑ]_ = ΔB_[ϑ]_ = 1.00 ± 0.02. In particular, the former result as to the Q values would point to a single association mode, at odd with the evidence from the combined use of chiro-optical and microcalorimetric data [11]. However, the apparent inconsistency between the finding that $Q_{1}$ = $Q_{2}$ and the existence of two $\theta \left(R_{j}\right)$ curves also from the new approach can be easily reconciled by considering the non-constant value of fitting parameter τ (see Figure 3a), which—for pectate—decreases by about 1/3 over the investigated range of R_j_ but, in turn, produces a much more dramatic difference between $\varphi_{1}$ and $\varphi_{2}$ as a function of R_j_; this can be neatly observed from Figure 3b.

(b) Alginate

**Figure 4:**
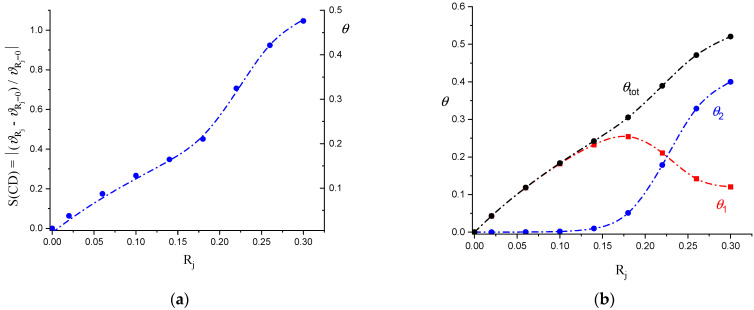
(**a**) CD, alginate—Dependence of the absolute value of the “specific” change of the molar ellipticity of sodium alginate as a function of the calcium-to-polymer molar ratio R_j_. Blue full circles, experimental data (bimodal); blue dash-dotted curve, fitting curve with the present method. (**b**) Fractions, alginate—fractions of chemically bound calcium ions by sodium alginate as a function of the molar ratio R_j_ calculated from the fitting of the CD data of panel (**a**). Red symbols, fractions of bonding mode 1, $\theta_{1}$, blue symbols, fractions of bonding mode 2, $\theta_{2}$.

In the case of alginate, the set of experimental parameters that can be used for testing the model is that of ${\left(S\left(\mathrm{CD}\right)\left(R_{j}\right)\right)}^{exp}$ only. The results of the procedure (whose details are given in the Appendix A, where the comparison between the results of minimization with a constant or a variable value of τ have also been reported) are the following: *Q*_1_ = 640, *Q*_2_ = 1270 (i.e., *Q*_1_ ≈ ½ · K_2_); ΔA_[ϑ]_ = 1.43, ΔB_[ϑ]_ = 2.18 (i.e., ΔA_[ϑ]_ ≈) ½ · ΔB_[ϑ]_), and $R_{j}^{Crit}$ = 0.25_2_. Mean squared error (MSE): 2.26 × 10^−4^; r.m.s. error = 0.15, with an estimated experimental error on ${\left(S\left(\mathrm{CD}\right)\left(R_{j}\right)\right)}^{exp}$ of 0.02. The experimental data points and the calculated (fitting) curve have been reported in Figure 4a, whereas the calculated values of the fractions of chemically bound calcium ions, $\theta_{1}$ and $\theta_{2}$, have been reported in panel (b) of Figure 4. The satisfactory convergence of the fit is demonstrated by the near identity of the (θ1θ2)RjCrit ratio, which is 0.50_2_ (being ${\left(\theta_{1}\right)}_{R_{j}^{Crit}}$ = 0.151, ${\left(\theta_{2}\right)}_{R_{j}^{Crit}}$ = 0.301), with the Q1Q2 ratio, which is 0.50_4_, namely to within 0.2%.

### 2.3. Comparison

#### 2.3.1. Fractions of Chemically Bound Calcium Counterions

**Figure 5:**
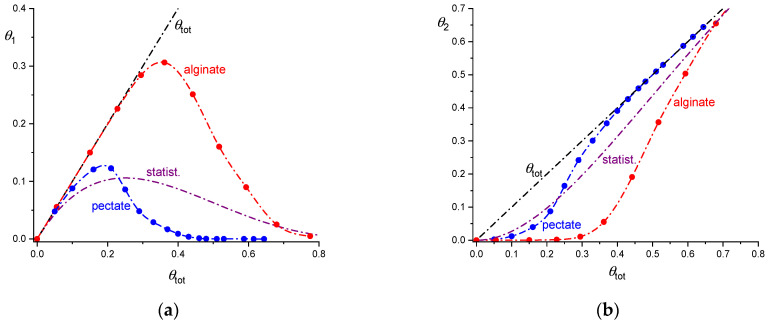
(**a**)—Theta 1. (**b**)—Theta 2. Dependence on the total fraction of chemically bound calcium ions ($\theta_{tot}$) of the fractions of bound ions of type-1 (panel (**a**)) and of type-2 (panel (**b**)), for pectate (blue filled circles) and alginate (red filled circles); the curves have been drawn to guide the eye. The purple curves represent the dependence on $\theta_{tot}$ of $\theta_{1}$ (panel (**a**)) and $\theta_{2}$ (panel (**b**)) calculated assuming a purely statistical filling up of sites with no interaction among nearest-neighboring sites (Equation (15)).

The dependence of the fractions of bound ions of type-1 and of type-2 has been reported in panel a and panel b of Figure 5, respectively, as a function of the total fraction of chemically bound calcium ions ($\theta_{tot}$), which is also the fractional extent of the transformation of the whole chemical bonding reaction (i.e., type-1 plus type-2). The curves representing the dependence of the two individual fractions calculated assuming a purely statistical filling up of sites with no interaction among nearest-neighboring sites have also been reported in both panels. It is useful to compare the experimental results with the calculated probability, $p_{n}$, based on the simple statistical model of filling up an array of *N* independent sites with *n* objects (without repetition):

(15) $$p_{n}=\left(1-\frac{n-1}{N}\right)\cdot \frac{n}{N}\cdot \left[\left[2+\left(N-2\right)\cdot \left(1-\frac{n}{N}\right)\right]\right]\cdot \left(1-\frac{n}{N}\right)$$

Moreover, n/N = $\theta_{tot}$.

Panel (a) shows that the $\theta_{1}$ values for both polyuronates increase beyond what was anticipated by the statistical model, much more so for alginate, before reaching the maximum. After that, a decrease of $\theta_{1}$ starts, which is much more dramatic for pectate than for alginate. (In the case of alginate, the wider range of prevalence of $\theta_{1}\left(R_{j}\right)$ over $\theta_{2}\left(R_{j}\right)$ is clearly to be traced back to the very large initial value of $\tau \left(R_{j}\right)$, which, only for ($R_{j}$) 0.25, tends to approach the less steeply changing values of pectate (see Figure 3a)). The decrease of $\theta_{1}$ finds a neat counterpart in the sigmoid-type increase of $\theta_{2}$ (see panel b). In other words, it is possible to describe the behavior of both polymers identifying an initial part on the $R_{j}$ axis in which there is a preference for singly bonded calcium ions (the “tilted” egg-box type), followed by an abrupt change of preference in favor of a bonding mode in which at least two (or more) calcium ions fill up two (or more) contiguous sites between facing chains (the “perfect” egg-box). This exactly matches the expected change of the inter-linker potential from repulsive to attractive in the theory of the association of two semiflexible polyelectrolytes by interchain linkers (“clusterization”) [22].

**Figure 6:**
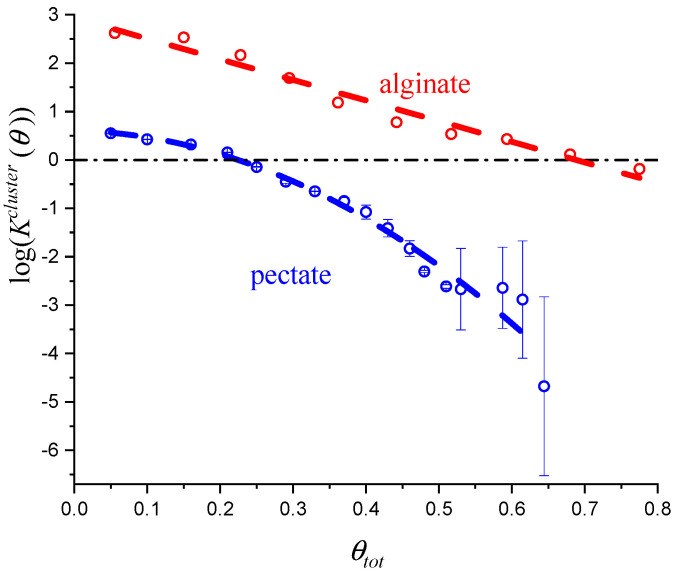
Dependence on the total fraction of chemically bound calcium ions, $\theta_{tot}$, of the logarithm of the clusterization constant, $K^{cluster}\left(\theta \right)$, as defined by Equation (16). Red symbols, alginate; blue symbols, pectate.

To better visualize such change, it can be convenient to define a “clusterization ratio” as:

(16) $$K^{cluster}\left(\theta \right)=\frac{{\left(\frac{\theta_{1}\left(\theta \right)}{\theta_{2}\left(\theta \right)}\right)}_{exper.}}{{\left(\frac{\theta_{1}\left(\theta \right)}{\theta_{2}\left(\theta \right)}\right)}_{statist.}}a=1,$$

where the symbols in the numerator refer to the experimentally determined fractions and those in the denominator to the corresponding fractions calculated from purely statistical approach. The logarithm of $K^{cluster}\left(\theta \right)$ is proportional to the inter-linker potential as defined by Borukhov et al. The calculated values of $\log(K^{cluster}\left(\theta \right))$ for both pectate and alginate have been reported in Figure 6. The difference between the two polyuronates is striking: whereas for the former one, the change of the sign of the potential takes place at about 22% of filling up (at $\theta_{tot}$ = 0.22_5_), for alginate, it takes place at almost 70% (at $\theta_{tot}$ = 0.68_3_)! Such a large difference is certainly rooted in different structural and/or macromolecular properties of the two polymers. To name some, there is the much larger compositional homogeneity of pectate vs. that of alginate and the much larger molar mass (MW) of alginate vs. pectate (see Section 4). As to the first point, in the case of pectate, the fraction of units different from (un-esterified) galacturonic acid was 0.10_6_, whereas in alginate the fraction of GG dyads is 0.53, thereby providing to the former polymer a favorable bias towards the formation of nearest-neighboring homogeneous sequences (i.e., $\theta_{2}$). On the other side, it is well-known that, for any percolation model of chain association, the formation of large clusters are favored by higher values of MW. In the case of alginate, the conformationally less demanding type-1 interchain links can play the ideal role of junctions of the highly branched, multi-chain structure. The scattering results that will be discussed in the last section show how large is the tendency of alginate to increase MW by the addition of calcium ions.

#### 2.3.2. Fractions of Condensed Calcium Counterions with Specific Affinity for Alginate: Evaluation of Their Fractions and of the Corresponding Thermodynamic Parameters

**Scheme 3:**
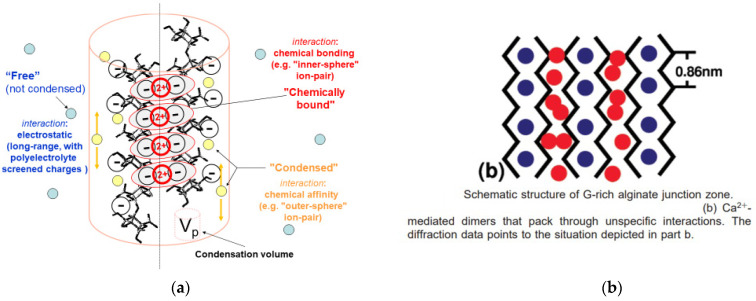
(**a**) Idealized representation of the three groups of calcium ions interacting with pectate (herein sketched in the “shifted egg-box” arrangement according to reference [20]) and alginate (see text). Redrawn with permission from Scheme 1 of reference [11] (© American Chemical Society, 2021). (**b**) Representation of chemically bound counterions (group (ii) in the “perfect” egg-box configuration (blue filled circles) and of “territorially” condensed counterions (group (i), red filled circles) according to Figure 6 of reference [21]. Reproduced with permission (© American Chemical Society, 2007).

The addition of calcium ions to the sodium salt form of a polyuronate in NaCl in the millimolar-to-molar range can be represented, from the polyelectrolyte standpoint, as a mixture of monovalent and divalent counterions in a system containing the monovalent salt of a polyanion. As highlighted at the onset of the section on “Pectate” herein above in paragraph A., extensive evidence over the latest decades has shown that divalent counterions can be—schematically albeit conveniently—divided into three groups: (i) “territorially” condensed counterions, specifically interacting with the polyanion due to some favorable “affinity”, entrapped in the “condensation volume” ${\bar{V}}_{p}$ surrounding the linear polyelectrolyte [13,14] but free to move within ${\bar{V}}_{p}$; (ii) chemically (albeit not covalently) bound counterions, bringing about local polymer charge annihilation; (iii) free counterions, in an exchange equilibrium with the former two types, but subject only to long-range electrostatic interactions with the partially shielded linear array of charges on the polyuronate. A detailed representation was given in Scheme 1 of reference [11], which has been redrawn in the present Scheme 3a,b. Groups (i) and (ii) as above described coincide with the “*disordered Ca^2+^ and Na^+^ cations (Figure 6b)*” and with “*Ca^2+^ cations … accommodated… in… pocket-like cavities*” as described by ref. [21], respectively.

The sum of the fractions of calcium counterions of type (i) and type (ii) correspond to the fraction of non-diffusible, or “osmotically inactive”, calcium counterions, $r^{osm}$:

(17) $$r^{osm}\left(R_{j}\right)=\left(1-\theta \left(R_{j}\right)\right)\cdot \;r_{C}\left(R_{j}\right)+\theta \left(R_{j}\right)\;\cdot \left(1-z_{j}\cdot \;\sigma^{0}\right)\cdot r_{D}\left(R_{j}\right)+\sigma^{0}\cdot \;\theta \left(R_{j}\right)=\;=C\left(R_{j}\right)+\;D\left(R_{j}\right)+\sigma_{tot}\left(R_{j}\right)={Ρ}^{cond}\left(R_{j}\right)+\sigma_{tot}\left(R_{j}\right)$$

where θ is the fraction of process transformation (i.e., of the filling of chemical bonding sites: $\theta =\theta_{tot}$), $\sigma^{0}$ is the calcium-to-repeating-unit stoichiometric ratio, $z_{j}$ is +2 for calcium, $r_{C}$ and $r_{D}$ are the fractions of condensed calcium counterions on the single-chain and the dimer (egg-box-linked) stretches of the polyuronate. ${Ρ}^{cond}$ (P is the capital Greek letter *rho*) is the fraction of condensed counterions of type (i) [9,11].

**Figure 7:**
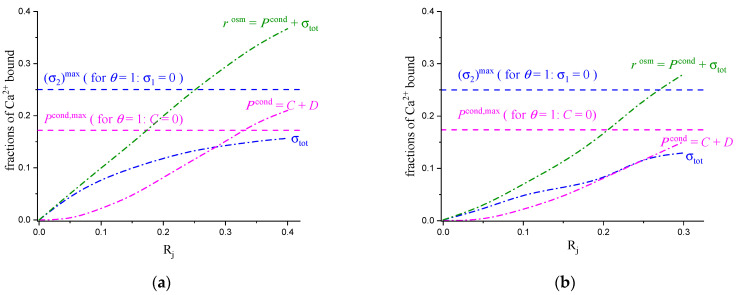
Dependence on R_j_ of the various fractions of bound calcium ions to: (**a**) pectate and (**b**) alginate. For definitions, see text.

In the case of pectate, the numerical evaluation of the fractions of counterions of both groups (i) and (ii) have been achieved [11]; the values have been replotted here in Figure 7a. For alginate, the treatment described in the previous paragraph produced the evaluation of chemically bound counterions, $\sigma_{tot}\left(R_{j}\right)$ (see Figure 7b). The complete solution of the problem of determining ${Ρ}^{cond}\left(R_{j}\right)$ and $\sigma_{tot}\left(R_{j}\right)$, together with both the enthalpic and the entropic components of both processed underlying the two types of binding, can be achieved following the demanding procedure described in reference [12]. To devise some alternative—faster—means for calculating ${Ρ}^{cond}\left(R_{j}\right)$, we resorted to introducing some hypotheses on the nature and consequences of the affinity interactions that determine the numerical value of $P^{cond}\left(R_{j}\right)$, applying them to the available dilatometric data of interaction between calcium and the two polyuronates (volume changes of “mixing”, $\Delta {\bar{V}}^{mix}$(R_j_)) [24]. As to the latter point, it is useful to recall the demonstrated assumption that the electrostatic component of the volume change from mixing a polyanion with cations ($\Delta {\bar{V}}^{el}$(R_j_)) in the presence of supporting electrolyte is negligible with respect to the “non-electrostatic” binding components ($\Delta {\bar{V}}^{non-el}$(R_j_): $\Delta {\bar{V}}^{el}$(R_j_) (($\Delta {\bar{V}}^{non-el}$(R_j_)) [25]. It is then possible to write:

(18) $$\Delta {\bar{V}}^{mix}\left(\mathrm{Rj}\right)\;\approx \Delta {\bar{V}}^{non-el}\;\left(R_{j}\right)=\Delta {\bar{V}}^{aff}\cdot \;{Ρ}^{cond}\left(R_{j}\right)+\theta_{1}\left(R_{j}\right)\;\cdot \;\Delta {\bar{V}}_{1}^{bond}+\theta_{2}\left(R_{j}\right)\;\cdot \;\Delta {\bar{V}}_{2}^{bond}$$

in which ${Ρ}^{cond}\left(R_{j}\right)$ must be independently calculated, and $\Delta {\bar{V}}^{aff}$, $\Delta {\bar{V}}_{1}^{bond}$ and $\Delta {\bar{V}}_{2}^{bond}$ are fitting parameters of the experimental volume changes of mixing: $\Delta {\bar{V}}^{aff}$ is the change of molar volume of specific affinity; $\Delta {\bar{V}}_{1}^{bond}$ and $\Delta {\bar{V}}_{2}^{bond}$ are the values of the change of molar volume of the strong chemical bonding of type-1 (“tilted” egg-box) and type-2 (“perfect” egg-box), respectively. The assumption that such parameters are constant, i.e., they do not depend on $R_{j}$, was already demonstrated [12].

The task of determining ${Ρ}^{cond}\left(R_{j}\right)$ requires the knowledge of a relation between ${Ρ}^{cond}\left(R_{j}\right)$ and an independent physical chemical quantity. To this end, Figure 4 of reference [12] shows and describes the parametric correlation between ${Ρ}^{cond}\left(R_{j}\right)$ (as $C\left(R_{j}\right)$ and $D\left(R_{j}\right)$) and the reduced Gibbs free-energy of affinity, $\frac{\Delta {\bar{G}}^{aff}}{RT}$. Much like $\Delta {\bar{V}}^{aff}$, $\Delta {\bar{G}}^{aff}$ is also supposed to be independent of R_j_. $\Delta {\bar{G}}^{aff}$ is, of course, not known a priori (nor is its components $\Delta {\bar{H}}^{aff}$ and $\Delta {\bar{S}}^{aff}$). However, it is herein proposed to arrive at a reasonable estimate of it, following two—only apparently demanding—hypotheses:

*i*  The affinity process is assumed to derive from desolvation only. The desolvation of both the condensed counterions and the polyion-charged groups is accompanied by positive changes of volume, enthalpy and entropy. The process is supposed to be *cratic* only, i.e., to stem essentially from the increase of the number of water molecules released from the hydration shells of the interacting ionic species (as to $\Delta {\bar{S}}^{aff}$), with the correlated rupture of several ion/dipole bonds (as to $\Delta {\bar{H}}^{aff}$). The observed positive volume change derives from the decrease of density (i.e., increase of ionic molar volume) of the released water molecules on passing from a condition of electrostriction to that of liquid water. This has been demonstrated by the positive values of the observed molar volume and enthalpy changes in various interactions of divalent ions with both synthetic polycarboxylates [26,27] and with polyuronates [24]. Therefore, both$\;\Delta {\bar{V}}^{aff}$ and $\Delta {\bar{S}}^{aff}$ and $\Delta {\bar{V}}^{aff}$ and $\Delta {\bar{H}}^{aff}$ are linearly proportional, i.e.,:

(19) $${\left(\Delta {\bar{S}}^{aff}\right)}_{i}={\left(k_{S}\right)}_{i}\cdot \;{\left(\Delta {\bar{V}}^{aff}\right)}_{i}$$

and

(20) $${\left(\Delta {\bar{H}}^{aff}\right)}_{i}={\left(k_{H}\right)}_{i}\cdot \;{\left(\Delta {\bar{V}}^{aff}\right)}_{i}$$

where each set of i-indexed values characterize each individual polyuronate species.

**Figure 8:**
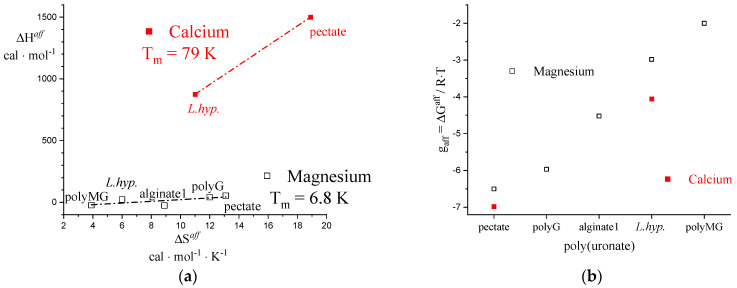
(**a**) Barclay–Butler plot—$\Delta {\bar{H}}^{aff}$ vs. $\Delta {\bar{S}}^{aff}$ —for different polyuronates interacting with Mg^2+^ ions (black open squares) and with Ca^2+^ (full red squares); (**b**) reduced Gibbs free-energy of affinity for the different systems of panel (**a**).

*ii*  The affinity interactions manifesting in the process of desolvation are intrinsically the same for all polyuronates/calcium systems, *i* (Barclay–Butler relationship [28]), namely they are characterized by a single value of T_m_ in the equation: Tm=(ΔH¯aff)i(ΔS¯aff)i . The Barclay–Butler correlation, which has found wide application in thermodynamic hydration studies for a long time, while [29], up to recent times [30], as well as in the thermodynamics of a solution of ionic polysaccharides [31]. On the experimental side, the results of reference [10], herein reported in Figure 8a, strongly support this hypothesis. In fact, they have been obtained in the parallel case of affinity interactions with magnesium ions exhibited by *L.hyp.* alginate, pectate, and by some related polyuronate systems. The ${\left(\Delta {\bar{H}}^{aff}\right)}_{i}$ vs. ${\left(\Delta {\bar{S}}^{aff}\right)}_{i}$ data show a very good linear correlation with a value of the slope as low as 6.8 K.

Ref. [11] provided the values of both $\Delta {\bar{H}}^{aff}$ and $\Delta {\bar{S}}^{aff}$ for the calcium/pectate system (see Table 3): $\Delta {\bar{H}}^{aff}$ = +1500 cal mol^−1^ and $\Delta {\bar{S}}^{aff}$ = +18.9 cal mol^−1^ K^−1^, with $\frac{\Delta {\bar{G}}^{aff}}{RT}$ = −7 and $T_{m}$ = 79 K. The combination of the two above hypotheses allows writing:

(21) (ΔS¯aff)alginate=(ΔS¯aff)pectate· (ΔV¯aff)alginate(ΔV¯aff)pectate

(22) (ΔH¯aff)alginate=(ΔH¯aff)pectate· (ΔV¯aff)alginate(ΔV¯aff)pectate

(23) (ΔG¯affRT)alginate=(ΔG¯affRT)pectate· (ΔV¯aff)alginate(ΔV¯aff)pectate

**Figure 9:**
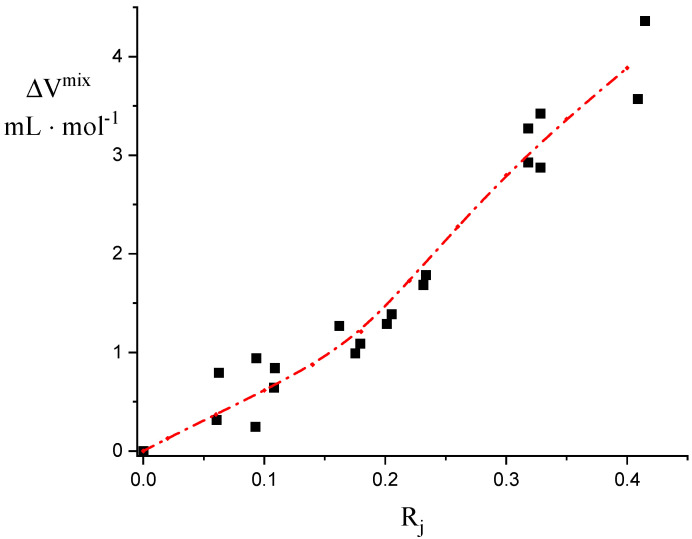
Volume change of mixing, $\Delta {\bar{V}}^{mix}$, calcium ions with *L. hyp.* alginate in aqueous 0.05 M NaClO_4_ at 25.0 °C, as a function of R_j_ (full black squares). Data taken from reference [24]. The red dash-dotted curve is the best-fit curve through the data according to Equation (18) (see text).

The value of ${\left(\Delta {\bar{V}}^{aff}\right)}_{alginate}$ is determined by the parametric fit of the experimental $\Delta {\bar{V}}^{mix}$ data points by an iterative process under the condition given by Equation (23). As a starting value of ${Ρ}^{cond}\left(R_{j}\right)$, it was decided to use that of pectate (see Figure 7), i.e., assuming $\frac{{\left(\Delta {\bar{G}}^{aff}\right)}_{alginate}}{RT}=\frac{{\left(\Delta {\bar{G}}^{aff}\right)}_{pectate}}{RT}$ = −7. Moreover, for simplicity, it was assumed that $\Delta {\bar{V}}_{1}^{bond}$ = $\Delta {\bar{V}}_{2}^{bond}$. The values of the fitting parameters were: $\Delta {\bar{V}}^{aff}$ = +7 mL · mol^−1^, $\Delta {\bar{V}}_{1}^{bond}$ = + 12 mL · mol^−1^, and $\Delta {\bar{V}}_{2}^{bond}$ = +12 mL · mol^−1^. The use of the determined value of $\Delta {\bar{V}}^{aff}$ in Equations (21) to (23) produced these results: $\Delta {\bar{S}}^{aff}\;$= +11 cal mol^−1^ · K^−1^, $\Delta {\bar{H}}^{aff}$ = +875 cal · mol^−1^, and $\frac{{\left(\Delta {\bar{G}}^{aff}\right)}_{alginate}}{RT}$ = −4.1. Using the latter value for the interpolation of the data in Figure 4 of Reference [12] returned: ${\left({Ρ}^{cond}\left(R_{j}\right)\right)}_{alginate}$= 0.66 $\cdot \;{\left({Ρ}^{cond}\left(R_{j}\right)\right)}_{pectate}$. The values of ${\left({Ρ}^{cond}\left(R_{j}\right)\right)}_{alginate}$ have been reported in Figure 7b, together with those of $\sigma \left(R_{j}\right)$. The limiting value of ${Ρ}^{cond,max}$ was calculated according to the procedure described in reference [11], assuming that calcium ions are preferentially bound by the GulA sequences, whose linear charge density value, ξ, was set equal to 1.64_0_, having taken the value of the average distance of the projections of the charges on the chain axis, *b*, as equal to 4.35 Å [32]; then ${Ρ}^{cond,max}$ = 0.17_4_. The new values of the fitting parameters of the experimental $\Delta {\bar{V}}^{mix}$ data points by use of Equation (18) were: $\Delta {\bar{V}}^{aff}$ = +7 mL·mol^−1^, $\Delta {\bar{V}}_{1}^{bond}$ = +9 mL·mol^−1^, and $\Delta {\bar{V}}_{2}^{bond}$ = +15 mL·mol^−1^. The result of the fitting has been reported in Figure 9.

Unexpectedly, the value of $\Delta {\bar{V}}^{aff}$ did not change from the previous run, indicating that convergence had been reached and also that the values of the other thermodynamic parameters of affinity were those already determined in the first run. They have all been reported in Table 3, together with the values of $\Delta {\bar{V}}_{1}^{bond}$ and $\Delta {\bar{V}}_{2}^{bond}$. The ${\left(\Delta {\bar{H}}^{aff}\right)}_{i}$ vs. ${\left(\Delta {\bar{S}}^{aff}\right)}_{i}$ data for alginate and pectate with calcium have also been reported in Figure 8a, clearly highlighting the much larger values of the thermodynamic parameters (particularly for $\Delta {\bar{H}}^{aff}$) for Ca^2+^ with respect to Mg^2+^. This is further evidenced by the plot of $\left(\frac{\Delta {\bar{G}}^{aff}}{RT}\right)$ for the different polyuronates, reported in Figure 8b). In the case of magnesium, pectate shows the highest affinity, followed by poly(guluronate), polyG. The alternating co-polymer polyMG exhibits the lowest affinity for the divalent ions, maybe not unexpectedly. This explains very nicely why both alginate 1 and *L.hyp.* alginates—with a mixed composition—show a much lower affinity for Mg^2+^ than pectate, the former alginate showing a higher affinity correlated with a higher GulA content (albeit only 70% vs. 65%). 

A comparative discussion of the thermodynamic parameters of the two polyuronates is given in next paragraph 4.

#### 2.3.3. Calculation of the Molar Enthalpy Changes of Chemically Bound Calcium Counterions

**Figure 10:**
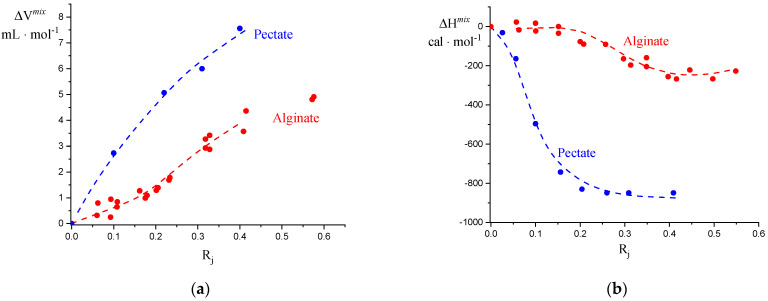
Volume (**a**) dilatometry and enthalpy (**b**) microcalorimetry change of mixing calcium ions with pectate (blue full circles) and *L.hyp*. alginate (red full circles) in aqueous 0.05 M NaClO_4_ at 25.0 °C, as a function of R_j_ (full black squares). Data taken from ref. [24].

Figure 10 reports in a comparative way the experimental dilatometric and microcalorimetric data upon mixing pectate and alginate with calcium ions. It is immediately apparent that, for both techniques, alginate and pectate show similar behavior, namely with positive values of molar volume change and negative values of molar enthalpy changes. However, in both cases, the values of alginate are lower than those of pectate. This is particularly relevant in the case of calorimetry; in fact, whereas the values of ${\left(\Delta {\bar{V}}^{mix}\right)}_{alginate}$ roughly amount to 60% of ${\left(\Delta {\bar{V}}^{mix}\right)}_{pectate}$, those of ${\left(\Delta {\bar{H}}^{mix}\right)}_{alginate}$ reach about only 25% of those of ${\left(\Delta {\bar{H}}^{mix}\right)}_{pectate}$. This points—once more—to underline the physical chemical relevance of enthalpy (i.e., internal energy, for condensed phases) to fine-tune structural differences between otherwise similar systems on one side and the high sensitivity of the microcalorimetric method on the other—i.e., the experimental—side.

**Figure 11:**
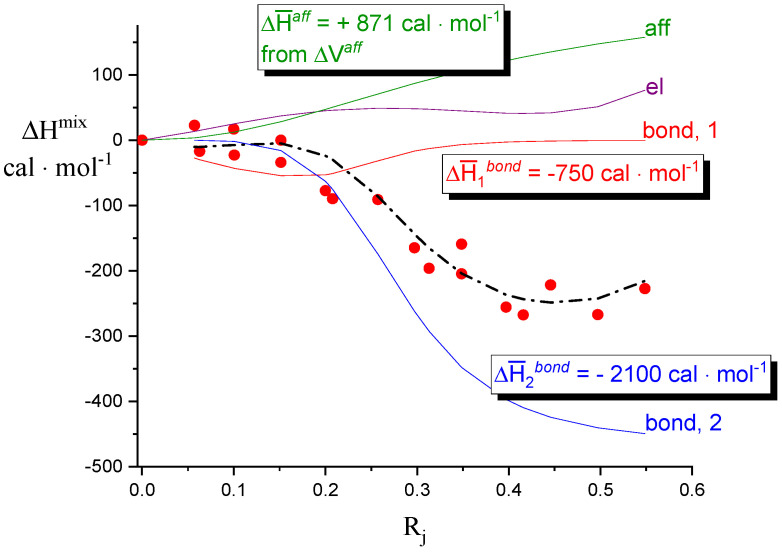
Enthalpy change of mixing calcium ions with *L. hyp*. alginate (red full circles) in aqueous 0.05 M NaClO_4_ at 25.0 °C, as a function of R_j_ (full black squares). Data taken from ref. [24]. “aff” stands for $\Delta {\bar{H}}^{aff}\cdot {Ρ}^{cond}\left(R_{j}\right)$, “el” for $\left(1-\theta \left(R_{j}\right)\right)\cdot H_{C}^{el}\left(R_{j}\right)+\theta \left(R_{j}\right)\cdot H_{D}^{el}\left(R_{j}\right)-H_{C}^{el}\left(R_{j}=0\right)$, “bond, 1” for $\theta_{1}\left(R_{j}\right)\cdot \Delta {\bar{H}}_{1}$ and “bond, 2” for $\theta_{2}\left(R_{j}\right)\cdot \Delta {\bar{H}}_{2}$, respectively.

Using the above determined value of $\Delta {\bar{H}}^{aff}$ and following the formalism described in refs. [11,12], the values of the (molar) enthalpy changes of the first and second chemical bonding modes have been determined (see Figure 11) using the equation:

(24) $$\Delta {\bar{H}}^{mix}\;\left(R_{j}\right)=\left(1-\theta \left(R_{j}\right)\right)\cdot \;H_{C}^{el}\left(R_{j}\right)+\theta \left(R_{j}\right)\cdot \;H_{D}^{el}\left(R_{j}\right)+\Delta {\bar{H}}^{aff}\cdot \;{Ρ}^{cond}\left(R_{j}\right)+\;+\theta_{1}\left(R_{j}\right)\;\cdot \;\Delta {\bar{H}}_{1}+\theta_{2}\left(R_{j}\right)\;\cdot \;\Delta {\bar{H}}_{2}-H_{C}^{el}\left(R_{j}=0\right)\;$$

where $H_{C}^{el}\left(R_{j}\right)$ and $H_{D}^{el}\left(R_{j}\right)$ are the R_j_-dependent values of the electrostatic enthalpy of the C and the D stretches, respectively, calculated according to the CC theory of linear polyelectrolytes. The fitting values are $\Delta {\bar{H}}_{1}^{bond}$ = −750 cal · mol^−1^ and $\Delta {\bar{H}}_{2}^{bond}$= −2100 cal · mol^−1^, respectively; they have been reported in Table 3.

#### 2.3.4. Thermodynamics of the Calcium/Polyuronate Interactions

The thermodynamic parameters accompanying the interaction of calcium ions with sodium pectate and sodium alginate reported in Table 3 point to both similarities and differences. As already stated, the overall interactions of both polyuronates clearly follow a similar pattern. The free energy of affinity is always favorable, due to the prevalence of the entropic over the enthalpic contributions, paralleled by—and rooted in—the positive volume changes. At variance with the affinity component, the calculated changes of both bonding modes are accompanied by negative (favorable) enthalpy changes.

However, even if presently unable to make any estimate of the entropic (and then of the Gibbs free-energy) component of both bonding modes, still the intensity of the collective interactions of alginate with calcium appear to be lower than that of the corresponding (affinity and bonding) interactions of pectate. The volume changes of bonding reveal a a very interesting similarity with the chiro-optical data. In fact, the monomodal increase of ${\left(S\left(\mathrm{CD}\right)\left(R_{j}\right)\right)}^{exp}$ for pectate produces two equal values of the scaling parameters: ΔA_[ϑ]_ = ΔB_[ϑ]_ = 1.00 ± 0.02. Likewise, the equally monomodal increase of the volume change, $\Delta {\bar{V}}^{total}\;\left(R_{j}\right)$, leads to $\Delta {\bar{V}}_{1}^{bond}$ = $\Delta {\bar{V}}_{2}^{bond}$ = +29 mL · mol^−1^, indicating that the conformational and the structural features of type-1 and type-2 modes of the chemical bonding of calcium by pectate are extremely superimposable, if not the same. At variance, both ${\left(S\left(\mathrm{CD}\right)\left(R_{j}\right)\right)}^{exp}$ and $\Delta {\bar{V}}^{mix}\;\left(R_{j}\right)$ curves of alginate are sigmoid and bimodal: in this case ΔA_[ϑ]_ = 1.43, ΔB_[ϑ]_ = 2.18 and $\Delta {\bar{V}}_{1}^{bond}$ = +9 mL · mol^−1^, $\Delta {\bar{V}}_{2}^{bond}$ = +15 mL · mol^−1^. The ratio of the former entities is 0.66 and that of the latter is 0.60, which is very close; this is particularly interesting if one recalls that they derive from completely independent experiments. (In spite of being devoid of any straightforward physical meaning, it still should be said that the ratio Q1Q2 for pectate is 0.98_3_, whereas that of alginate is 0.50). In conclusion, even lacking the whole thermodynamic picture of the bonding interactions of alginate, it seems sound to conclude that the “tilted” egg-box mode of the bonding of this polymer shows a significant difference from the “perfect” egg-box mode. Pectate shows a high similarity of some intrinsic aspects of the two modes of bonding as to both desolvation ($\Delta {\bar{V}}_{1}^{bond}$, $\Delta {\bar{V}}_{2}^{bond}$) and to relative conformational change (ΔA_[ϑ]_, ΔB_[ϑ]_); the large difference between $\Delta {\bar{H}}_{1}^{bond}$ and$\;\Delta {\bar{H}}_{2}^{bond}$ can reasonably be traced back to the cooperative effects of the propagation of the “perfect” structure rather than to an intrinsic difference between type-1 and type-2 modes of chemical bonding. 

#### 2.3.5. Macromolecular Properties

**Figure 12:**
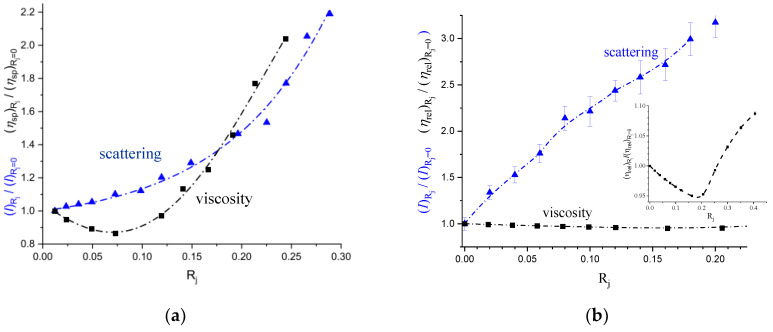
Dependence on the R_j_ of the ratio of the experimental property at R_j_ over the same property at R_j_ = 0; full blue triangles: scattering intensity at 90°, *I*; full black squares: specific viscosity, ·_sp_. (**a**) pectate (data from refs. [33,34]; (**b**) *L. hyp.* alginate (data from ref. [18]).

Whole-chain properties may be of great help in extending the information already provided by the thermodynamic properties on the overall effects of calcium binding by polyuronates. In particular, viscosity can explain a lot about the hydrodynamic volume of the polymers (extension), whereas light-scattering can parallel that with high sensitivity to changes in molar mass (association). The ratio of the reduced specific viscosity and of the scattering intensity at a value R_j_ over the corresponding value at R_j_ = 0 for a sample of sodium pectate and for a sample of sodium alginate have been reported in Figure 12a,b, respectively. (In this case it was chosen to use the data on the pectate sample from reference [33,34] as reproduced in reference [11], which a had a molar mass much closer to that of alginate to ensure a better comparison of whole-chain properties). In both cases, the two sets of data follow the same pattern: ηRjηRj=0 initially decreases upon increasing R_j_ (for alginate, this is more neatly seen from the inset of panel (b) to eventually show an increase). This behavior has induced erroneous interpretations as to an alleged initial intramolecular bonding of calcium [35,36]. Sticking to one single experimental technique is never a good choice; in particular, it is not if the technique is sensitive primarily to dimensions (volume), and only indirectly to molar mass, like viscosity. In fact, the parallel behavior of scattering, IRjIRj=0, neatly shows that the average molar mass of both pectate and alginate start immediately rising after the initial additions of calcium, as a proof of all-time intermolecular calcium bonding.

In the next final part, attention will be given to *L. hyp*. alginate, given the attention that pectate has already received in previous work [11,12]. Although the high accuracy of the viscometric measurements warrants full reliability to the V-shaped behavior shown in the inset of panel (b), it is useful to consider the numerical values of the scattering and viscosity curves of alginate. Whereas in the former case, the relative increase at the highest value of R_j_ is more than 200%, the relative decrease of ηRjηRj=0 is not more than 5%, followed by a final relative increase of only 10%. It means that, in front of a massive chain association induced by even tiny amounts of calcium, the variation in the hydrodynamic volume of the soluble associating species changes only slightly.

**Figure 13:**
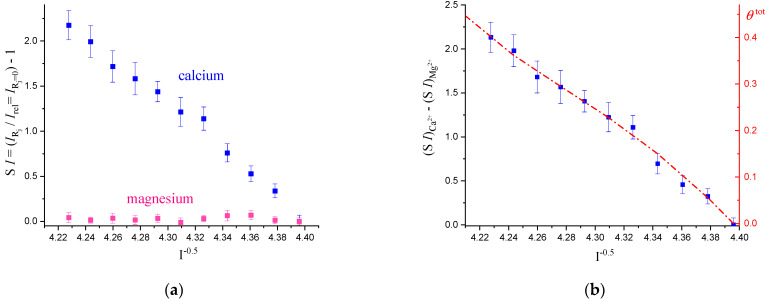
(**a**) Dependence of the relative scattering intensity increase at 90°, S *I*, on the reciprocal of the square root of the ionic strength, I, for the addition of Ca^2+^ ions (blue full squares) and Mg^2+^ ions (pink full squares) to *L. hyp.* alginate in aqueous NaCl 0.05 M. (**b**) Blue full squares: dependence on the reciprocal of the square root of the ionic strength, I, of the difference of the relative scattering intensity at 90°, S *I*, between the data with Ca^2+^ ions and those with Mg^2+^ ions of panel (**a**); red dash-dotted curve: dependence on the reciprocal of the square-root of the ionic strength, I, of the total fraction of chemically bound Ca^2+^ ions to *L. hyp.* alginate (r.h.s. ordinate).

When dealing with the whole-chain properties of polyelectrolytes, the most theoretically sound compositional variable is the inverse of the square root of the ionic strength, I, which represents the scaling dependence of long-range electrostatic interactions on the Debye length. Figure 13a reports the relative scattering increase dependence, $S\;I\;\left(R_{j}\right)$, on I^−0.5^ upon the addition to sodium alginate of calcium and magnesium ions, respectively. The latter data keep being practically zero (the average value is 0.031 ± 0.048), with no indication of dependence on I^−0.5^, pointing to no change in molar mass. On the contrary, an increase of the ionic strength by the addition of calcium ions produces the already ascertained increase of MW. The data points of panel b) of Figure 13 are the values of the difference between the values of $S\;I\;\left(R_{j}\right)$ measured in the presence of calcium minus the corresponding values in the presence of magnesium (for reasons that will become apparent in discussing the next paragraph on viscosity). The curve drawn through the points represents the total fraction of chemically bound calcium ions, taken from Figure 4b. The excellent fit of the experimental data by the $\theta_{tot}\left(I^{-0.5}\right)$ curve unambiguously traces back the observed increase of molar mass to the overall chemical bonding of Ca^2+^. In full analogy with Equation (13) used for fitting the CD data, one can write for the scattering ones:

(25) $$S\left(I\right){\left(R_{j}\right)}_{calcium}-S\left(I\right){\left(R_{j}\right)}_{magnesium}=\Delta A_{I}\;\cdot \theta_{1}\left(R_{j}\right)+\Delta B_{I}\;\cdot \theta_{2}\left(R_{j}\right)$$

Still using the $\theta_{1}\left(R_{j}\right)$ and $\theta_{2}\left(R_{j}\right)$ data from Figure 4b, one determines: $\Delta A_{I}$ = 5.3_3_ and $\Delta B_{I}$ = 9.2_5_. Although $\Delta B_{I}$ is about 74% larger that $\Delta A_{I}$, pointing to the more important role of “perfect” egg-box structures in forming the inter-polymer cross-links, the value of $\Delta A_{I}$ still indicates that the chain association brought about by “tilted” egg-box structures is already quite large.

**Figure 14:**
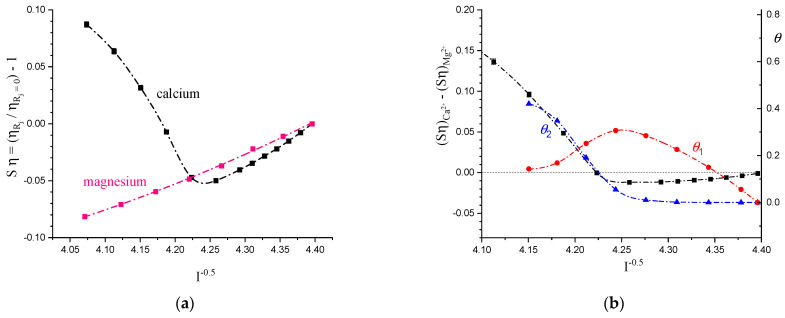
(**a**) Dependence of the relative increase of specific viscosity, S·, on the reciprocal of the square root of the ionic strength, I, for the addition of Ca^2+^ ions (black full squares) and Mg^2+^ ions (pink full squares) to *L. hyp.* alginate in aqueous NaCl 0.05 M. (**b**) Black full squares: dependence on the reciprocal of the square root of the ionic strength of the difference of the relative increase of specific viscosity, S·, between the data with Ca^2+^ ions and those with Mg^2+^ ions of panel (**a**); full symbols and dash-dotted curves: dependence on the reciprocal of the square root of the ionic strength of the fractions of type-1 (red) and type-2 chemically bound Ca^2+^ ions to *L.hyp.* alginate, respectively (r.h.s. ordinate).

The use of I^−0.5^ as the independent variable is almost the rule when dealing with the viscosity of polyelectrolytes [37,38], given the strong effect of polyion charge shielding on chain dimensions brought about by an even comparatively limited change of ionic strength. The $S\eta \left(R_{j}\right)$ values of sodium alginate upon the addition of calcium and magnesium ions are reported in Figure 14a. In the case of Mg^2+^ ions, the behavior is very close to the linear one expected on theoretical grounds [37]. The slight upward curvature upon decreasing I^−0.5^, though, is also theoretically predicted inasmuch as the addition of divalent ions brings about a modest but finite decrease of the effective linear charge density of the polyelectrolyte, ξ_eff_. This progressively brings the experimental points from the initial line to move along a surface in a (Sη, I^−0.5^, ξ_eff_) space, towards lines whose dSηdI−0.5 slope decrease upon decreasing ξ_eff_. This effect is even more pronounced in the case of Ca^2+^ ions down to about I^−0.5^ = 4.25. However, the explanation given for magnesium does not hold for calcium. In fact, the latter ion clearly exhibits a much higher tendency to interact with alginate than magnesium (see Figure 8b), thereby producing a higher reduction of ξ_eff_; as a consequence, and the expected Sη(I^−0.5^) data points should be still negative but less negative than those of Mg^2+^. The explanation is the same as that given for the similar behavior of the calcium/pectate system [11], namely that, in general, the lateral association of two chains produces a decrease—albeit small—of the total hydrodynamic volume and hence a decrease of Sη. Upon further calcium addition beyond I^−0.5^ = 4.25, the system undergoes the progressive formation of multi-chain associates, which brings about a massive increase of the total hydrodynamic volume and hence the sudden upturn of Sη(I^−0.5^).

To trace back the whole Sη vs. I^−0.5^ curve to the chemical bonding of Ca^2+^ ions, we resorted to writing an expression similar to that reported in Equation (25):

(26) $$S\eta {\left(R_{j}\right)}_{calcium}-S\eta {\left(R_{j}\right)}_{magnesium}=\Delta A_{\eta }\;\cdot \theta_{1}\left(R_{j}\right)+\Delta B_{\eta }\;\cdot \theta_{2}\left(R_{j}\right)$$

The determined best-fit parameters were: $\Delta A_{\eta }$ = −0.15_4_ and $\Delta B_{\eta }$ = 0.13_4_. The small negative value of $\Delta A_{\eta }$ neatly confirms the very small reduction of the hydrodynamic volume induced by the formation of the “tilted” egg-box structure (as indicated by the correlation with $\theta_{1}$). Nevertheless, what is clear-cut is the correlation between the increase of $S\eta {\left(R_{j}\right)}_{calcium}-S\eta {\left(R_{j}\right)}_{magnesium}$ and the formation of “perfect” egg-box structures (i.e., $\theta_{2}$), in parallel with the second, larger, increase of $S\;I\;\left(I^{-0.5}\right)$. As a final comment on the viscosity, the comparison between the corresponding numerical values of the scaling parameters of type-1 and type-2 of the bonding between scattering and viscosity confirms the previous statement about the much lower response of the latter technique with respect to the former one to macromolecular, whole-chain variations: the values of ΔA and ΔB for the scattering data are one full order of magnitude larger than those from viscosity; those from CD somewhat fall in between them.

A final comment on the success of the new method described in this work for the calculation of the fractions of chemically bound divalent ions, $\theta_{1}\left(R_{j}\right)$ and $\theta_{2}\left(R_{j}\right)$, comes from the fact that the experimental data as a function of $R_{j}$ from three different techniques can be very well fitted with the values of the fractions provided by the new calculation method, with the only provision of two constant values of the scaling parameters for each technique—one for each bonding type.

## 3. Conclusions

The new method of calculation of the fractions of chemically bound calcium counterions, initially based on the experimental CD data but soon after further extended to encompass light-scattering and viscosity data, allowed for providing—also for alginate—the conceptual tool for reaching a complete thermodynamic picture of the interaction of that algal polysaccharide with the important gelling ion, much like as it had previously done for pectate. It can be easily anticipated, however, that the present approach can be generalized to other multivalent ions as well, theoretically or practically. The found similarities of behavior of the two polymers are striking:

- Ca^2+^ counterions are preferentially accumulated as territorially condensed counterions around the polyanions thanks to a “specific affinity” for the carbohydrate polymer moiety;
- such a “cloud” of crowded calcium ions contributes stabilizing the further strong chemical bonding in conformationally specific intermolecular sites, widely known as “egg-boxes”;
- the strong chemical bonding (entailing charge annihilation, albeit not the formation of covalent bonding) develops through two sequential steps. The former one—type-1—involves the formation of an imperfect—or “tilted”—mononuclear egg-box, which upon further calcium ions addition transforms into the sequence of nearest neighboring “perfect” egg-boxes—type-2;
- the change of the molar mass of both pectate and alginate upon increasing concentration of calcium shows that the formation of calcium interchain links starts from the beginning, with no “induction-concentration”, possibly deriving from intramolecular calcium bonding.

However, differences exist between alginate and pectate:

1. the attainment of the conformational ordering of pectate is the same for both the “tilted” and the ”perfect” egg-box, at variance with alginate, for which such an attainment of conformational order is quite lower for the type-1 mode than that for the type-2 one;
2. type-1 bonding mode of alginate is favored with the respect to type-2 for a range of the growing calcium concentration much wider than that of pectate. In both cases, however, the behavior of the linker formation growth perfectly parallels that predicted by the model of Borukhov et al.;
3. all thermodynamic parameters of calcium interaction with pectate indicate a stronger affinity in the case of pectate than for alginate.

The last above point clearly addresses the need to extend the above described approach to alginates having a limit composition as to the constituent uronates, to trace back the found differences to their molecular basis.

## 4. Materials and Methods

The pectate (poly(galacturonate), polyGal) sample used in the reported experiments was already described; in particular, the fraction of units different from (un-esterified) galacturonic acid (GalA) was 0.10_6_ [11,39,40] The relative molecular mass of the sodium salt form of the equivalent repeating unit (r.u.), M_r.u._, was M_r.u._ = 222.8_29_; the polymer concentration, C_p_, is expressed in equivalent·L^−1^ (eq·L^−1^). The value of the number-average relative molecular mass, ${\bar{M}}_{n}$, determined by membrane osmometry was: ${\bar{M}}_{n}$ = 2.1 · 10^4^ (i.e., the molar mass was 21 · 10^3^ kg·mol^−1^) [11].

Sodium alginate isolated from *L. hyperborea* stipe (relative molar mass, MW∼130,000) was provided by FMC Biopolymers (Norway) [18]. Alginate 1 was the sodium salt form of algal alginate purchased from Fluka (relative molar mass, MW~52,000) [24,41]. The compositional properties of both samples are given in Table 3.

**Table 3:** Chemical composition and intrinsic viscosity of alginate from *L. hyperborea* ^a^.

Sample	F_G_	F_M_	F_GG_	F_GGG_	F_GM,MG_	F_MM_	[η] (dL/g) ^b^
*L. hyperborea* alginate	0.65	0.35	0.53	0.49	0.12	0.23	6.41 ± 0.02
Alginate 1	0.45	0.55	0.30	n.a.	0.15	0.40	n.a.

^a^ F_G_ and F_M_ denote the fraction of alginate consisting of guluronic acid (GulA, G) and mannuronic acid (ManA, M), respectively. F_GG_ and F_GGG_ indicate the fraction of alginate consisting of guluronic acid in blocks of dimers and trimers, respectively, whereas F_MM_ indicates the fraction of alginate consisting of mannuronic dyads. F_GM,MG_ indicates the fraction of alginate consisting of mixed sequences of guluronic and mannuronic acid. ^b^ Solvent: NaCl 0.1M, 20 °C.

The chloride (Cl^−^) and/or the perchlorate (ClO_4_^−^) salt forms of sodium (Na^+^), calcium (Ca^2+^), and magnesium (Mg^2+^) were purchased from Sigma-Aldrich (St. Louis, MO, USA).

All experimental methods whose results are reported in this paper have been described in detail in the Supporting Information of Donati et al. [11].

## Figures and Tables

**Table 1:** Thermodynamic parameters of the affinity (upper part, A.) and of chemical bonding (lower part, B.) interactions of Ca^2+^ ions with pectate (l.h.s.) and with *L.hyp*. alginate (r.h.s.).

Pectate			Alginate	
**A**. Affinity				
$\Delta {\bar{V}}^{aff}$	12	mL·mol^−1^	$\Delta {\bar{V}}^{aff}$	7
$\Delta {\bar{S}}^{aff}$	18.9	cal·mol^−1^ K^−1^	$\Delta {\bar{S}}^{aff}$	11.0
$\Delta {\bar{H}}^{aff}$	1500	cal·mol^−1^	$\Delta {\bar{H}}^{aff}$	875
$\Delta {\bar{G}}^{aff}$	−4135	cal·mol^−1^	$\Delta {\bar{G}}^{aff}$	−2411
g^aff^	−7.0	(g^aff^ = $\Delta {\bar{G}}^{aff}/RT)$	g^aff^	−4.1
**B**. Bonding				
$\Delta {\bar{V}}_{1}^{bond}$	29	mL·mol^−1^	$\Delta {\bar{V}}_{1}^{bond}$	9
ΔA[𝜗]	1.00	-	ΔA[𝜗]	1.43
$\Delta {\bar{V}}_{2}^{bond}$	29	mL·mol^−1^	$\Delta {\bar{V}}_{2}^{bond}$	15
ΔB[𝜗]	1.00	-	ΔB[𝜗]	2.18
$\Delta {\bar{H}}_{1}^{bond}$	−1704	cal·mol^−1^	$\Delta {\bar{H}}_{1}^{bond}$	−750
$\Delta {\bar{H}}_{2}^{bond}$	−7817	cal·mol^−1^	$\Delta {\bar{H}}_{2}^{bond}$	−2100

**Table 2:** Limiting behavior of $\varphi_{1}$ and $\varphi_{2}$ as a function of the independent variable $R_{j}\;$ and of the parameter τ.

For			And			Then	
						$\varphi_{1}\left(R_{j}\right)$	$\varphi_{2}\left(R_{j}\right)$
$R_{j}$	=	$R_{j}^{Crit}$	τ	Finite value		1/2	1/2
$R_{j}$	=	0	τ	→	∞	1	0
$R_{j}$	=	0	τ	=	0	1/2	1/2
$R_{j}$	=	0	τ	→	- ∞	0	1
$R_{j}$	→	∞	τ	Finite value		0	1

**Figure A1:**
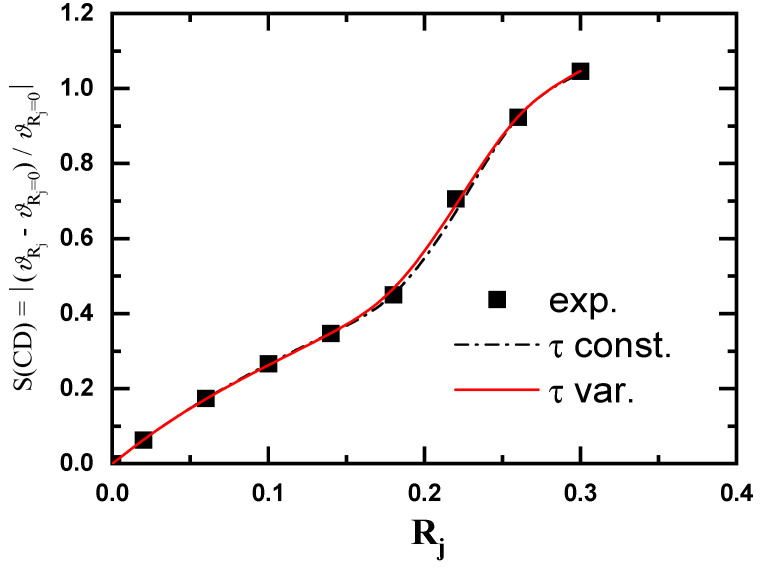
Best fit of the experimental data using Equation (13) for alginate from *L. hyperborea*. Black line represents the best fitting using a constant value of τ, while the red curve represents the best fitting with τ = τ ($R_{j}$), using the values of the dotted curve of Figure 3a.

## Appendix A

The results of the minimization procedure using the new method on the CD data are reported in Figure A1. Two approaches were followed. In the first one, τ is kept constant for all the $R_{j}$ values. In the second one, τ was allowed to vary with $R_{j}$, exactly as shown by Figure 3a. After testing several equations, a four-parameter logistic-like equation was found to give the best results:

(A1) τ=1−A−D1+(RjC)B

being A = 0.5, B = 2, C = 0.3, and D = 4 for the test case of pectate.

For alginate, the values were: A = 3, B = 4.81, C = 0.301, and D = 15.7.

For both the reported cases, the fitting is very good, pointing to the reliability of the model developed. The results of the minimization of the parameters are reported in Table A1; the same general trend can be noticed regardless of the approach used. In Figure A1, the comparison between the calculated curve with a constant value of τ (black curve) and that with variable τ (red curve) shows that the latter one is slightly better than the former one. The numerical values of the fitting parameters for the two cases of the τ constant and variable are reported in Table A1.

**Table A1:** Parameters obtained from the minimization of Equation (13) using τ as defined in the column headings.

Parameter	Constant τ	Variable τ
$\Delta A_{\vartheta }$	1.26	1.43
$\Delta B_{\vartheta }$	1.72	2.18
Q_1_	759	640
Q_2_	1488	1270
$R_{j}^{Crit}$	0.22_5_	0.25_2_
τ	11.1	-
